# Pathological Findings and Distribution of Pandemic Influenza A (H1N1) 2009 Virus in Lungs from Naturally Infected Fattening Pigs in Norway

**DOI:** 10.1155/2011/565787

**Published:** 2011-12-20

**Authors:** Mette Valheim, Hans Gamlem, Britt Gjerset, Anna Germundsson, Bjørn Lium

**Affiliations:** ^1^Department of Laboratory Services, Norwegian Veterinary Institute, Pb 750 Sentrum, N-0106 Oslo, Norway; ^2^Department of Health Surveillance, Norwegian Veterinary Institute, Pb 750 Sentrum, N-0106 Oslo, Norway

## Abstract

The Norwegian pig population was considered free from influenza A virus infections until the first case of porcine pandemic influenza A (H1N1) 2009 virus infection in October 2009. Human to pig transmission of virus was suspected. Unusual lung lesions were observed in fattening pigs, with red, lobular, multifocal to coalescing consolidation, most frequently in the cranial, middle, and accessory lobes. The main histopathological findings were epithelial degeneration and necrosis, lymphocyte infiltration in the epithelial lining and lamina propria of small bronchi and bronchioles, and peribronchial and peribronchiolar lymphocyte infiltrations. Infection with pandemic influenza A (H1N1) 2009 virus was confirmed by real-time RT-PCR and immunohistochemical detection of influenza A virus nucleoprotein in the lesions. This investigation shows that natural infection with the pandemic influenza A (H1N1) 2009 virus induces lung lesions similar to lesions described in experimental studies and natural infections with other swine-adapted subtypes of influenza A viruses.

## 1. Introduction

Infections with swine-adapted subtypes of influenza A virus are enzootic in most pig-producing countries and cause a highly contagious respiratory disease in pigs [1, 2]. The pathological changes are restricted to the respiratory tract with necrotizing bronchitis and bronchiolitis and multifocal lobular purulent bronchopneumonia as the main lesions [3].

In April 2009, a novel strain of influenza A H1N1 virus, now known as pandemic influenza A (H1N1) 2009 (pandemic H1N1 2009 virus), affecting humans was detected in Mexico and USA [4]. The new strain was shortly afterwards reported to be transmitted from humans to pigs, and the following few weeks infection in pigs was reported from Argentina, Canada, Australia, Northern Ireland, Ireland, and the United States [5–7].

The first porcine case of pandemic H1N1 2009 virus in Norway was detected in the beginning of October 2009 [8]. A farm staff member had shown influenza-like illness and had tested positive for pandemic H1N1 2009 virus in the same period as a sow showed signs of respiratory disease, and human to pig transmission was suspected. In the following three months, infection with pandemic H1N1 2009 virus was widespread and detected in 91 Norwegian pig herds [9].

The Norwegian pig population is relatively small, totaling 2,500 herds and with annual production of 1,5 million fattening pigs. Up to October 2009 the population was free from influenza A virus infection (subtypes H1N1 and H3N2) as judged by a serological surveillance program running since 1997 [10]. Moreover, porcine respiratory and reproduction syndrome (PRRS) virus and *Mycoplasma hyopneumoniae* are not present in the Norwegian pig population [10, 11].

As the first herds tested positive for pandemic H1N1 2009 virus, lungs from slaughtered pigs with unusual lung lesions were collected for pathological examination and virus detection. This paper describes the pathological findings and virus distribution in lungs from slaughtered fattening pigs from herds naturally infected with pandemic H1N1 2009 virus.

## 2. Materials and Methods

### 2.1. History and Animals

In December 2009 an unusual type of lung lesions in pigs was reported by meat inspectors at some abattoirs in Norway. Lungs from pigs in four different herds were collected at two different abattoirs in the south eastern Norway and sent to the Norwegian Veterinary Institute for pathological and virological examination.

In three of the herds (herd 1, 2, and 3), there was no history of clinical respiratory disease, and the lungs were selected for examination only due to the unusual type of lung lesions observed at the meat inspection. From these three herds, lungs from 9, 10, and 4 pigs, respectively, were examined. Herds 1 and 3 were finishing herds, while herd 2 was an integrated herd.

In the fourth herd (herd 4), a finishing herd, the pigs had coughed more than usual. Since the farmer and his family had shown influenza-like symptoms, the herd had been tested for pandemic H1N1 2009 virus infection by examination of 20 nasal swabs 18 days before this group of pigs had been slaughtered. All the samples were positive. Lungs from eight slaughtered pigs were submitted for examination.

### 2.2. Gross Pathological Examination

Gross lesions in the lungs and available distal part of the trachea and pathological findings in the tracheobronchial lymph nodes were recorded.

### 2.3. Samples from the Lungs

In total, one sample from the trachea and nine lung samples, three from each of the cranial lobes and one from the middle lobe, the accessory lobe, and the right caudal lobe, respectively, were collected. All samples were collected from the ventral parts of the lung. In the cranial lobes samples from both peripheral and central areas were collected. In addition, one sample from a tracheobronchial lymph node was collected. Swabs were collected for influenza virus nucleic acid detection by real-time RT-PCR, from the trachea and one main bronchus from each examined pig.

### 2.4. Histopathological and Immunohistochemical Examination

Samples for histopathological examination were fixed in 10% buffered formalin for 24 h and then transferred to 70% ethanol. The samples were processed omitting the formalin bath, and 3-4 *μ*m sections were cut and stained with hematoxylin and eosin.

To confirm an infection with influenza A virus in the lung tissue, an immunohistochemical examination using monoclonal antibodies directed against influenza A virus nucleoprotein (Anti-Influenza A (NP), Statens Serum Institut, DK) was performed [12]. Briefly, a secondary antibody conjugated to a horse radish peroxidase-labeled polymer (Dako EnVision+ System-HRP, 3-amino-9-ethylcarbazole (AEC)) was used. From each examined lung one sample from each of the two cranial lobes and one sample from a caudal lobe were examined. One section from the trachea and one from a tracheobronchial lymph node were also included in the examination.

Immunohistochemical examination for porcine circovirus type 2 (PCV2) antigens was performed using a monoclonal antibody kindly provided by Dr. Gordon Allan (The Virology Department, Veterinary Sciences Division, Faculty of Agriculture and Food Sciences, The Queens University of Belfast, Northern Ireland). Briefly, trypsin-treated antigens were labelled with the monoclonal antibody and visualised with a streptavidin-biotin, alkaline phosphatase method with fast red as substrate. Three sections from cranial, middle, and caudal lung lobes from each pig were examined.

### 2.5. Real-Time RT-PCR for Virus Nucleic Acid Detection

Total RNA was extracted from 200 *μ*L of the sample material using the automatic extraction instrument NucliSens easyMag (bioMérieux Norge AS, Oslo, Norway) according to the manufacturer's instruction. Detection of influenza A virus was performed as described by WHO Collaborating Centre for influenza at Centers for Disease Control and Prevention (Atlanta, USA) [13]. Positive samples were tested by a specific real-time RT-PCR detecting the HA gene of the pandemic H1N1 2009 virus as described by the Robert Koch-Institute, Germany [14]. Amplification was performed on a Stratagene Mx3500P (LaJolla, CA, USA) with superscript III platinum one-step quantitative RT-PCR system (Invitrogen, Paisley, UK). In addition, samples were analyzed at the National Veterinary Institute, Technical University of Denmark using an in-house real-time RT-PCR for detection of pandemic H1N1 2009 virus nucleic acids (Statens Serum Institut, Denmark, unpublished). One RT-PCR-positive sample was defined as sufficient to confirm a herd as positive for pandemic H1N1 2009 virus.

### 2.6. Bacteriological Examination

One tissue sample from a cranial lobe from each lung was submitted for bacteriological examination.

## 3. Results

### 3.1. Gross Findings

**Figure 1:**
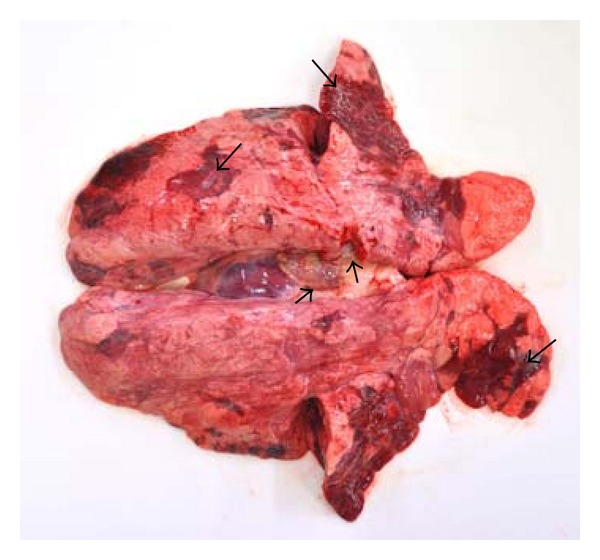
(Lung) Dorsal view. Dark red, multifocal atelectasis or mild consolidation (large arrows) primarily in cranial and middle lobes, but also in caudal lobes. The tracheobronchial lymph nodes were slightly enlarged (small arrows).

**Figure 2:**
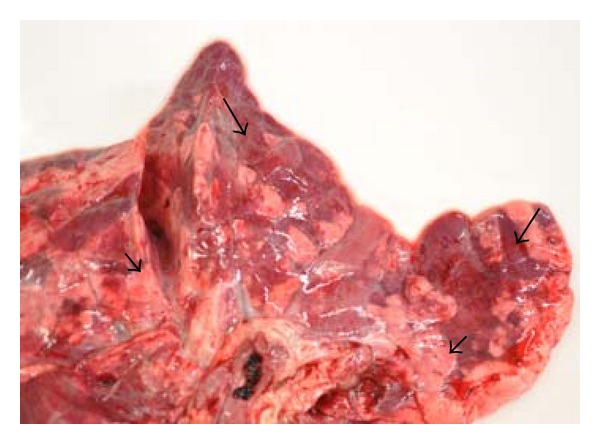
(Lung) Cranial and middle lobe. Lobular, multifocal dark red atelectasis or mild consolidation (large arrows) alternating with pink, emphysematous lobules (small arrows).

The gross examination of the lungs from herds 1, 2, and 4 revealed multifocal to coalescing areas with mild consolidation and dark red discoloration (Figure 1). The lesions showed a lobular distribution, and between the dark red lobuli, pale pink, often emphysematous tissues were seen (Figure 2). The lesions were most frequently observed in the cranial, middle, and accessory lung lobes, but scattered lesions were also present in the caudal lobes. The lesions were more pronounced in the ventral part of the lungs. Scarce to moderate amounts of mucus or mucopurulent exudate were observed in the bronchi. The tracheobronchial lymph nodes were firm, slightly to moderately enlarged with a pale, moist cut surface. 

In these herds two lungs showed different types of lesions. In one lung from herd 1, distinct, grey-red, firm areas were observed in the cranial lobes, while in one lung from herd 2 (pig no. 10) a grey-yellow, firm lung tissue was seen primarily in the cranial lobes and abundant mucopurulent exudate was present in the bronchi and on the cut surface. The tracheobronchial lymph nodes showed prominent enlargement.

In herd 3 only two lungs showed similar but less widespread lobular, dark red lesions as described in lungs from herds 1, 2, and 4, while one lung showed a diffuse, grey consolidation with mucopurulent exudate on the cut surface and moderate enlargement of the tracheobronchial lymph nodes. In the fourth lung, there were scattered areas with atelectasis, but no other findings were recorded.

### 3.2. Histopathological Findings

**Figure 3:**
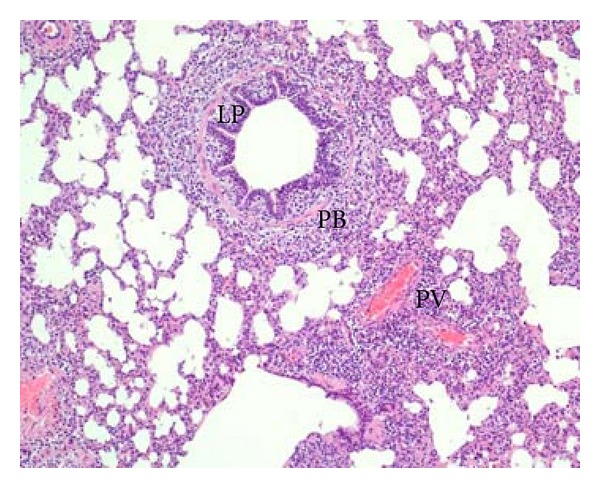
(Lung) Acute phase. Moderate infiltration of lymphocytes in the lamina propria (LP) in a bronchiole and peribronchiolar (PB) and perivascular (PV) lymphocyte infiltration. HE, 10x.

The main histopathological findings in all the examined lungs in the four herds were moderate to intense infiltration of leukocytes in the lamina propria and epithelium of small bronchi and bronchioles and mild to prominent peribronchiolar, peribronchial, and perivascular lymphocyte infiltrates (Figure 3). The lesions were multifocal and showed a lobular distribution and were observed in the majority of the examined sections, but they were most prominent in the cranial and central parts of the lungs. Moderate to severe bronchiolar and bronchial lesions were also observed in areas with no gross lesions as well as in one lung from herd 3 with no gross pathological registrations.

Necrotic bronchial and bronchiolar epithelium and attenuation of the epithelial lining with flattening of the epithelial cells were seen. Varying amounts of granulocytes, sloughed epithelial cells, cell debris, and mucus were present in the lumina of many bronchioles and small bronchi.

In the lungs with mild to prominent peribronchiolar and perivascular infiltrates of lymphocytes, the infiltrates often extended into the adjacent interalveolar and interlobular septa. In several affected areas a moderate number of small- to moderate-seized peribronchiolar and peribronchial lymphoid follicles were observed.

Areas of atelectasis were a significant finding in the majority of the examined lungs. Varying numbers of leukocytes were seen in the partially or totally collapsed alveoli.

In two lungs (one lung from herd 1 and one lung from herd 3) with a more prominent consolidation and abundant mucopurulent exudate, the inflammatory response was dominated by granulocytes in bronchiolar and alveolar lumina and protein-rich fluid was observed in the alveolar lumina and in the interstitial tissue.

**Figure 4:**
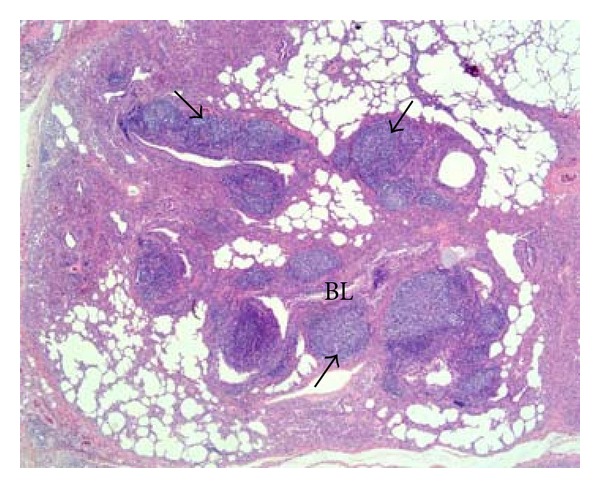
(Lung) Convalescence phase. Prominent peribronchial lymphoid hyperplasia with multiple large lymphoid follicles (arrows) that sometimes seemed to compress the lumina of small bronchi (BL) and bronchioles. HE, 10x.

The histopathological lung lesions in the pigs from herd 4 showed great similarity to what is described for lungs in herds 1–3, but the peribronchial and peribronchiolar lymphocytic infiltrations were more prominent with formation of multiple, large lymphoid follicles (Figure 4). The large lymphoid follicles often seemed to compress the bronchiolar lumina. Atelectasis was also an important finding in these lungs.

 Examination of the trachea showed scarce to moderate infiltration of lymphocytes in the epithelial lining and in the lamina propria. In the tracheobronchial lymph nodes mild to moderate follicular hyperplasia and infiltration of neutrophils and eosinophils in sinuses were observed.

### 3.3. Immunohistochemical Examination for Virus Detection

Influenza A virus nucleoprotein was detected by immunohistochemical examination in lungs from herds 1–3, while in the lungs from herd 4 no virus protein was detected. The virus protein was detected in five of nine (44%) lungs from herd 1, in nine of ten (90%) lungs in herd 2, and in three of four (75%) lungs in herd 3. In the majority of the virus protein-positive lungs, nucleoprotein was detected in two or three of the three examined sections.

**Figure 5:**
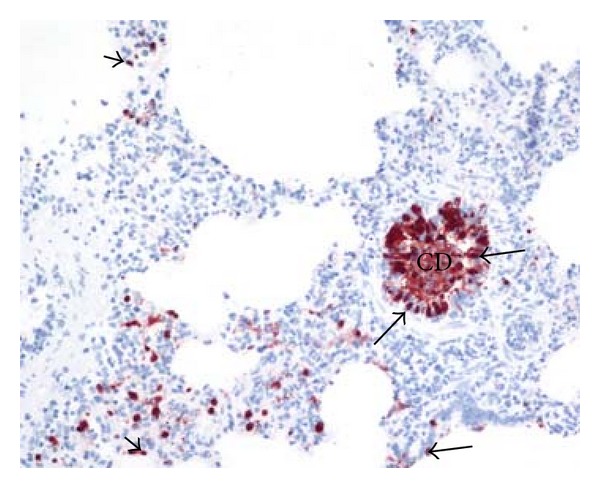
Abundant influenza A virus nucleoprotein was detected in epithelial nuclei (large arrows) and in luminal cellular debris (CD) in bronchioles and small bronchi and in the nuclei (small arrows) in pneumocytes lining the alveolar septa. Immunohistochemistry AEC, counterstain hematoxylin, 20x.

**Figure 6:**
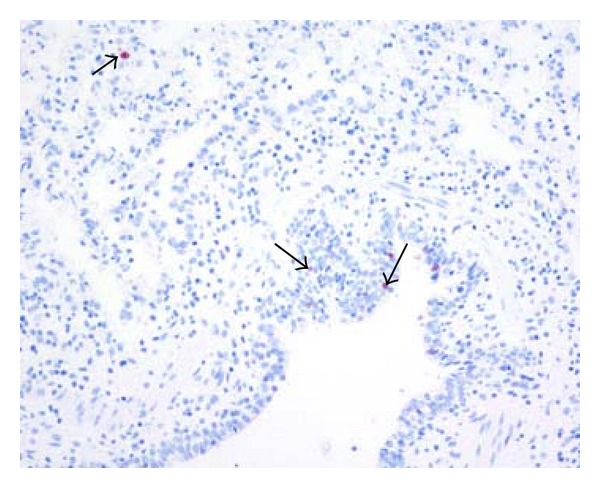
(Lung) Sparse influenza A virus nucleoprotein was detected in few nuclei (large arrows) in bronchial and bronchiolar epithelial cells and in few nuclei (small arrow) in pneumocytes in the parenchyma. Immunohistochemistry AEC, counterstain hematoxylin, 20x.

In lungs from six pigs originating from all the three virus protein-positive herds, moderate to abundant virus protein was detected multifocally in the bronchiolar and small bronchial epithelia and in luminal cellular debris (Figure 5). In addition virus protein could be detected in the nuclei of pneumocytes lining the interalveolar septa and in the cytoplasma of cells in alveolar lumina. In the remaining virus protein-positive lungs, only few scattered virus protein-positive nuclei in epithelial cells in bronchi, bronchioles and interalveolar septa were detected (Figure 6). Virus protein was not detected in samples from the trachea and tracheobronchial lymph node.

The immunohistochemical examination for PCV2 antigens in the three lung sections from all the 31 pigs was negative.

### 3.4. Real-Time RT-PCR for Detection of Pandemic H1N1 2009 Virus Nucleic Acids

Pandemic H1N1 2009 virus nucleic acids were found in the respiratory tract (tracheal and bronchial swabs) in eight out of eight (100%) pigs tested in herd 1, in nine of ten (90%) pigs, and all four (100%) pigs, tested from herds 2 and 3, respectively, by real-time RT-PCR. Virus nucleic acids were not detected in the tracheal or bronchial swabs from the five pigs tested from herd 4.

### 3.5. Bacteriological Examination

The bacterial examination of lung tissue revealed growth of* Pasteurella multocida *and *Arcanobacterium pyo*genes from one lung (herd 2, pig no. 10), sparse to moderate growth of *Streptococcus* spp. (*S*. *dysgalactiae equisimilis*, *S. porcinus*,* S. suis, and Streptococcus* sp.) from 11 lungs, and sparse *Staphylococcus* spp. from two lungs. From the remaining lungs, sparse mixed nonpathogenic bacteria or no bacteria were isolated.

## 4. Discussion

The pandemic H1N1 2009 virus infection induced multifocal bronchointerstitial pneumonia in fattening pigs. The lesions could be related to the pandemic H1N1 2009 virus infection in the majority of the pigs since influenza A virus nucleoprotein was demonstrated in the lesions. The examination for PCV2 antigens was negative, and the herds were free from PRRS virus and *M. hyopneumoniae.* Since the Norwegian pig population has been free from influenza A virus infections, this is the first description of influenza-A-virus-induced lung lesions in Norwegian pigs.

Lobular pneumonia with major lesions in small bronchi and bronchioles with detection of virus mostly in epithelial cells showed great similarity to what is described for experimental infection with the pandemic H1N1 virus and infections with other adapted subtypes (H1N1, H3N2, and H1N2) of influenza A virus [2, 15].

The real-time RT-PCR examination of swabs from the trachea and one main bronchus detected more influenza virus-infected pigs (21 of 22; examined; 95%) than did the immunohistochemical examination of lung parenchyma, trachea, and tracheobronchial lymph node (17 of 23 examined; 74%). Whilst RT-PCR has higher sensitivity, the use of immunohistochemistry shows the distribution of the virus protein within the lungs. The usefulness of using both methods is illustrated in Figure 5 which shows the accumulation of virus-infected cells within bronchioles and small bronchi. Thus samples taken for RT-PCR in the trachea will comprise cells that have drained from the entire lung and collected into the upper airway tract.

In herds 1–3 (representing an acute phase of infection), a mild to prominent lymphoid infiltration was observed around small bronchi and bronchioles and blood vessels in the examined lungs. In addition, few to a moderate number of small- to moderate-sized lymphoid follicles were observed, an indication of subacute lesions. In the majority of these lungs, influenza A virus nucleoprotein was detected (Figures 5 and 6). The duration of the infection in these lungs most probably has varied between five to ten days prior to slaughter, since experimental infections of pigs with high health status with pandemic H1N1 2009 virus have shown that the virus cannot be detected 10-11 days after infection [15–17]. In eleven of the virus protein-positive lungs, only few virus-protein-positive cells (Figure 6) were observed despite widespread lesions indicating that these pigs were about to combat the virus infection.

In the fourth herd (representing a convalescent phase), where the pigs were slaughtered 18 days after pandemic H1N1 2009 virus nucleic acids had been detected in the herd, neither virus nucleic acids nor influenza A virus nucleoprotein could be detected in the lungs. A prominent peribronchiolar and peribronchial lymphoid hyperplasia were seen in these lungs (Figure 4). This intrapulmonary, lymphoid hyperplasia may contribute, together with the lymph node follicular hyperplasia, to the rapid and strong serological response detected in pigs infected with pandemic H1N1 2009 virus [17].

The significance of the bacteria isolated was evident only for one lung, where a mixture of *Pasteurella multocida* and *Arcanobacterium pyogenes *was isolated from firm lung tissue with abundant mucopurulent exudate in the bronchi. In two more lungs with grayish consolidation in the cranial parts and extensive diffuse consolidation, respectively, *Streptococcus porcinus* was isolated from the lung tissue, but the importance of this bacterium for the observed pathological lesions is uncertain.

Despite the multifocal lung lesions in herds 1–3, there was no history of typical clinical signs associated with swine influenza (nasal discharge, coughing, fever, and reduced appetite). A modest monitoring of fattening pigs may explain why mild symptoms might have been overlooked. Moreover, increased frequency of coughing had been observed in herd 4.

The main differential diagnoses of lobular pneumonia in the Norwegian pig population are pneumonia caused by *Mycoplasma* species other than *M. hyopneumoniae*, as *M. flocculare* and porcine circovirus type 2. Histological examination and detection of pathogens in the lesions are necessary to discriminate between the different pneumonias.

Since 1997, testing for antibodies against swine influenza virus by hemagglutination inhibition test has been included in the Norwegian surveillance and control programme for specific virus infections in swine herds. In 1998, 17 pigs from one herd tested positive for antibodies against H3N2 virus. Follow-up investigations, however, showed no further spread of the virus among the pigs in the herd, and the virus was never established in the Norwegian pig population [18]. This indicates that the pigs had been infected with a human adapted H3N2 virus.

Serological examination of the pig population in 2010 revealed that about 40% of the Norwegian pig herds were seropositive for pandemic H1N1 2009 virus [18]. This reflects a rapid dissemination of the pandemic H1N1 2009 virus in the susceptible Norwegian pig population. Pandemic H1N1 2009 virus contains gene segments of both human and swine influenza origin which makes the virus replicate well in both humans and pigs without any further adaptation to a new host [4]. This has probably been important for the introduction of this influenza virus to the Norwegian pig population.

## 5. Conclusion

Natural infection with the pandemic influenza A (H1N1) 2009 virus in a susceptible pig population produces lung lesions similar to which are described in experimental studies with this virus and from natural outbreaks of infections with other swine-adapted subtypes of influenza A viruses. The distribution of the virus in the lung tissue also showed marked similarity to what is described for other swine-adapted influenza A viruses.
